# Age-specific nomograms for follicle stimulating hormone and anti-Mullerian hormone: A pilot study in Ile-Ife, Nigeria

**Published:** 2016-12

**Authors:** Omoladun Temitope Okunola, Olusegun Kayode Ajenifuja, Morebise Olabisi Loto, Afolabi Salawu, Oluseyi Stephen Omitinde, Joel Akande, Elizabeth Oke

**Affiliations:** 1 *Department of Obstetrics and Gynecology, Obafemi Awolowo University Teaching Hospitals Complex, Ile-Ife, Nigeria.*; 2 *Department of Obstetrics and Gynecology, Obafemi Awolowo University, Ile-Ife, Nigeria.*; 3 *Department of Chemical Pathology, Ladoke Akintola University of Technology Teaching Hospital, Ogbomoso, Nigeria.*

**Keywords:** *Ovarian reserve*, *Follicle stimulating hormone*, *Anti-Müllerian hormone*, *Nomogram*, *Reference values*

## Abstract

**Background:**

Assessment of ovarian reserve is one of the steps in the management of infertile couples. Follicle Stimulating hormone (FSH) and anti-Müllerian hormone (AMH) are commonly used ovarian reserve markers in Africa. However, there is paucity of age-specific reference values for FSH and AMH among the African population.

**Objective:**

This study aimed at conducting a pilot study for generation of age-specific nomograms for FSH and AMH among fertile women in Ile-Ife, Nigeria.

**Materials and Methods:**

A pilot cross-sectional study that involved 65 fertile women within the age range of 18-45 yr were prospectively and consecutively recruited from November 2014 to January 2015. Peripheral blood samples were taken for basal serum FSH and random serum AMH. The samples were processed using enzyme linked immunosorbent (ELISA) assays.

**Results:**

Age-specific FSH nomogram showed a gradual increase which became steeper at age 35 yr with an average yearly increase of 0.2 IU/L in basal serum FSH, while age-specific AMH nomogram showed a peak at 25 yr and then; an average yearly decrease of 0.11 ng/ml in random serum AMH from 25 yr.

**Conclusion:**

The age-specific nomograms generated by this pilot study suggest that AMH may be an earlier marker of reduced ovarian reserve; which if validated by future multicenter population based studies may facilitate counseling of women on their reproductive potentials.

## Introduction

Ovarian reserve (OR) refers to the number and quality of oocytes that, at any given age, are available to produce a dominant follicle late in the follicular phase of menstrual cycle (1). The determination of the ovarian reserve is one of the important steps in investigating an infertile couple. Ovarian reserve is used to predict the remaining reproductive lifetime, response to ovarian stimulation and likely success of assisted reproductive techniques such as in vitro fertilization (2-4). 

Ovarian reserve tests include age, basal follicle stimulating hormone (FSH), luteinizing hormone (LH), basal oestradiol, clomiphene citrate challenge test (CCCT), anti-Müllerian hormone (AMH) and Inhibin B (4-6). Others are exogenous follicle stimulating hormone tests (EFORT) and gonadotropin releasing hormone agonist stimulation test (4-6). Ultrasound parameters for determining ovarian reserve include antral follicular count and basal ovarian volume (4-6). Ovarian vascularity and ovarian biopsy have also been tried but have been considered not to add any information to antral follicular count and other non-invasive tests (4, 5).

In most people, fertility potential starts declining after the age of 30 and moves downward rapidly thereafter, essentially reaching zero by the mid-40s (6). The ideal parameter to estimate ovarian reserve should be easily measurable, minimally invasive, inexpensive, and have good predictive value for the outcome being assessed (5). FSH is the most commonly used among the ovarian reserve tests (7).

 FSH is an indirect marker of ovarian reserve; it is produced in the anterior pituitary gland in response to estrogen secreted by the follicles (1). It is well studied and validated; providing a level of confidence to physicians (1, 7, 8). It has been in clinical practice for many years but has been found to have a lot of drawbacks (8). FSH exhibits inter-cycle and intra-cycle variability (9). Additionally, elevated level of FSH is a late indicator of decreased fertility potential (7). However, it is still being used in developing countries like Nigeria.

AMH, which is produced exclusively by the granulosa cells has distinct advantages over other ovarian reserve tests (ORTs) and has been found to be a better marker of ovarian reserve (1). AMH does not exhibit significant intra-cycle variability; hence, it can be measured on any day of the cycle (2, 10). Also, it exhibits less inter-cycle variability (10). It is believed to show declining ovarian reserve early in reproductive life cycle of a woman (1). It is presently being used in clinical practice to predict response to ovarian hyperstimulation and IVF success (11).

Researchers have documented age-specific reference values for FSH, estrogen, AMH and antral follicular count. Despite the fact that basal serum FSH levels is frequently being used to assess ovarian reserve, interpretation of its values is still not universal due to paucity of age-specific reference values among the African population (12). 

In 2014, Grisendi *et al* created age-specific reference values for basal serum FSH among regularly menstruating women in Italy (12). Also, la Marca *et al* created an age-specific normogram for AMH among regularly menstruating women in the reproductive age group (13). Age-specific nomograms for FSH and AMH in our environment are yet to be available. Age-specific nomograms will assist in screening women for early ovarian ageing; which is present in 10% of the population, predicting reproductive age, predicting chances of conception in women desirous of pregnancy and counseling of those desirous of delaying childbearing (14). 

Therefore, the aim of this study was to conduct a pilot study for generating age-specific nomograms for FSH and AMH.

## Materials and methods

**Study participants**

This was a cross-sectional study that involved sixty-five fertile women recruited from the Gynaecology Clinic and General Outpatient Department of the Obafemi Awolowo University Teaching Hospitals Complex, Ile-Ife (November 2014-January 2015). This study was approved by the Ethics and Research Committee of Obafemi Awolowo University Teaching Hospitals Complex, Ile-Ife. (Ethical clearance certificate number ERC/2014/05/01). At recruitment, an informed consent was obtained from each participant and the mobile telephone numbers were also documented.

The inclusion criteria were age between 18 -45 yr, regularly menstruating with cycle length between 21 and 35 days, proven natural fertility with at least one pregnancy carried to term within the preceding 2 years; and each pregnancy arising spontaneously following unprotected sexual intercourse within 1 year. Women with history, radiological and biochemical parameters suggestive of polycystic ovarian disease (PCOD), diabetes mellitus and thyroid diseases and those used hormonal contraceptives within the last 2 yr were excluded from the study. 

The study proforma was then filled to document the demographic and gynecological information. The outcomes of the study were basal serum FSH and random serum AMH. A venous blood sample was taken for serum AMH measurement. The women were instructed to alert the investigator at the onset of her next menstrual cycle in order to make arrangement for collection of day 3 FSH sample. Peripheral blood samples (5 mls) were collected through a venopuncture by a doctor for each of FSH and AMH assay. 

Samples were collected into plain sterile sample bottles and left to stand for 1 hr for clot retraction and then centrifuged for 10 min at 5,000 rpm. Serum was then separated into another unheparinised sterile sample using a pipette. The serum was then stored in a -20^o^C freezer until analyzed within 3 wk. The samples were transported to the laboratory in ice packs.

**FSH and AMH assays**

The samples were processed in duplicates at the Metabolic Research Laboratory of Ladoke Akintola University of Technology teaching hospital, Ogbomoso. Control sera were included in each batch. Serum FSH was quantified with Follicle Stimulating Hormone Test System manufactured by Monobind Inc, USA using the direct enzyme linked immunosorbent (ELISA) assay according to the manufacturer’s manual. 

The precision of the assay was 0.134 mIU/ml. AMH was quantified with Human AMH ELISA kit manufactured by Span Biotech Ltd, Hong Kong using a double-antibody sandwich ELISA according to manufacturer’s manual. The sensitivity of the assay was 0.01 ng/ml. After incubation, the absorbance was read at 450 nm within 30 min using microplate ELISA reader (LT 4000).

**Statistical analysis**

Convenience sampling was used to determine the sample size for this study. Sixty-five fertile women were recruited and included in the analysis. Data was analyzed using Stata version 13 manufactured by StataCorp. Means and standard deviations were used to summarize continuous variables. Scatter diagrams were plotted to depict the relationships between age, FSH and AMH. Nomograms were generated from natural log transformed values using the 10^th^, 50^th^ and 90^th^ centiles among the women. 

## Results

**Patients’ characteristics**

Out of the 89 women considered for recruitment, a total of 65 women that satisfied the inclusion criteria and consented were recruited into the study from November 2014 to January 2015. The basal serum FSH levels, random serum AMH levels and age were normally age distributed. The mean age, BMI and parity of the fertile group were 31.28±6.23, 26.31±4.68 and 1.95±1.08 respectively while the mean age, BMI and parity of the infertile group were 32.02±4.67, 26.03±5.24 and 0.48±0.97 respectively. Majority of the participants belong to the Yoruba ethnic group in Nigeria; 93.8% of them were from the Yoruba women while others belong to other ethnic groups in Nigeria.

**AMH and FSH nomograms**

**Figure 1:**
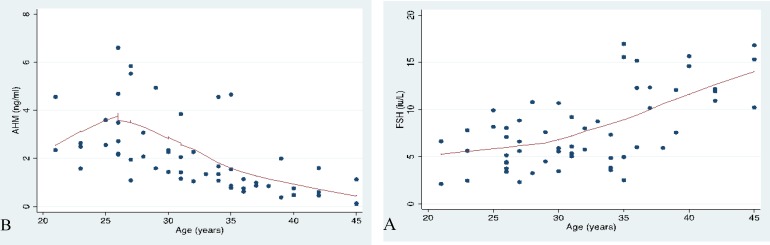
Relationship between the hormones and age in Ile Ife, Nigeria. A) Follicle stimulating hormone; B) Anti-mullerian hormone.

**Figure 2:**
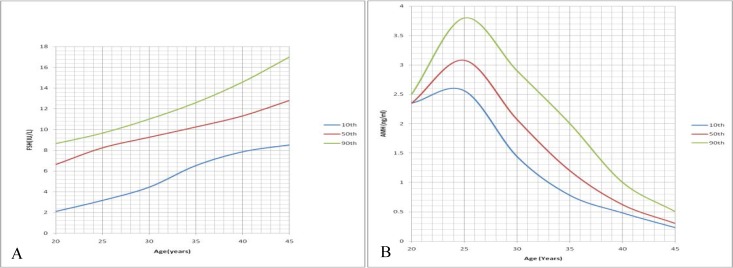
Age-specific nomograms for follicle stimulating hormone and anti-mullerian hormone in Ile, Ife, Nigeria. A) Follicle stimulating hormone. B) Anti-mullerian hormone

Figure 1 showed the relationship between age and FSH and AMH respectively. Nomograms were constructed using the 10^th^, 50^th^ and 90^th^ percentiles. The FSH normogram showed a steady increase in FSH levels as the age increases; which became steeper from age 35 upwards (Figure 2). There was an average yearly increase of 0.2 IU/L in the basal serum FSH. There was however an initial rise in serum AMH nomogram from 20-25 yr. It then showed a steady decrease in AMH levels with advancing age (Figure 2). There was an average yearly decrease of 0.11 ng/ml in the random serum AMH.

## Discussion

Age specific FSH nomogram showed a gradual increase in basal serum FSH with increasing age and this was comparable to findings of other researchers that generated nomogram (12). Grisendi *et al* also established a linear relationship between FSH and age; an increase of 1 IU every 9 yr among the Italian women (12). This was different from what was observed in this study in which there was an increase of 1 IU every 5 yr; genetics may be responsible for this. This difference may also be due to disparity in sample size.

Age-specific AMH nomogram peaked at 25 yr; and this was comparable to other studies by Lie Fong and Kelsey (15, 16). It thereafter decreases with age till the end of the reproductive years (15, 16). There was a gradual decrease in random serum AMH from 25-45 yr. La marca *et al* also demonstrated steady decline in AMH with age using a nomogram generated with 5^th^, 25^th^, 50^th^, 75^th^ and 95^th^ percentile (17). This finding was contradictory to findings from other studies that fertility starts declining at 35 yr (18, 19). The FSH nomogram showed decline at age 35 yr while the AMH nomogram showed decline at age 25 yr. This could corroborate findings that AMH shows decline in ovarian reserve earlier than FSH; and thus may be an earlier marker of reduced ovarian reserve.

The strengths of this study include the prospective study design and being an attempt to generate a nomogram from population specific data. This pilot study has clearly demonstrated the feasibility of national study to generate FSH and AMH nomograms among African women. The sample size for this study may be small but the variables were normally distributed; and thus can be used to generate a nomogram. 

A limitation of the study was the fact that the hormone levels were not correlated with number of oocytes retrieved, pregnancy rate or live birth. A large multicentered study will be necessary to generate nomogram that will be applicable to the Nigerian population and determine other factors associated with deviation from the FSH and AMH normogram. Such study should ensure good rapport and confidentiality between the investigators and participants in order to ensure that the true age of the women are reported.

The age-specific nomograms to be generated and validated by future multicentered studies may facilitate counseling of the women on their reproductive potentials based on population specific data derived from this environment. Also, random serum AMH showed decline in ovarian reserve earlier than basal FSH in the reproductive years; and this further suggests that AMH may be an earlier marker of diminishing ovarian reserve. 
